# The Parkes Museum of Hygiene

**Published:** 1888-06

**Authors:** 


### THE PARKES MUSEUM OF HYGIENE.

Her Royal Highness, the Duchessof Albany, presented on Saturday last, at the Parkes Museum, certificates to the ladies who had passed an examination following a course of lectures on domestic hygiene given by Dr. Schofield on behalf of the National Health Society. Sir Douglas Galton, who opened the proceedings, spoke of the need which was felt by friends of hygiene and workers in sanitation at this critical point, when further progress of the sanitary education of the nation required earnest and concentrated effort, and expressed a strong belief that, if all the different sanitary and health associations were combined, ample funds for the endowment of a grand national college of hygiene, with affiliations in the chief localities of the empire, would be at once available. * * * * * *
A letter from Miss Florence Nightingale, expressing her deep sympathy with the objects of the Museum, especially with the ceremony that day, was read; it contained the following passage: "Without women there can be no domestic hygiene. The first principles and work of sewerage, water-supply, and ventilation must, without the housewife, almost remain a dead letter. But let her be practically interested in how to keep air, earth, and water pure, and to admit light in her house, and the health and life-giving machinery is complete."—*British Medical Journal, May* 12.

---

### TREATMENT OF MERCURIAL STOMATITIS.

Dr. E. de Renzi, of Naples, reports that he has treated several cases of mercurial stomatitis with corrosive sublimate with satisfactory results. He used this drug because he thought that a powerful antiseptic was indicated in a disease characterized by putrefactive processes. The sublimate was used as a mouth-wash, 25 centig. to 1000 grams of water being used in two days. On the first day the foul smell of the breath was removed, and within three days the redness and swelling of the mouth had diminished very much. The patients were cured on the fifth day as a rule.—*Rivista Clinica e Terapeutica,* February, 1888.

